# Effect of Compound Fertilizer on Foxtail Millet Productivity and Soil Environment

**DOI:** 10.3390/plants13223167

**Published:** 2024-11-11

**Authors:** Yanyan Duan, Chenyang Wang, Lizhi Li, Ruihua Han, Xiao Shen, Genlan Han, Jiang Wang, Mengen Nie, Xinlei Zhou, Huiling Du, Xiangyang Yuan, Shuqi Dong

**Affiliations:** 1 College of Agriculture, Shanxi Agricultural University, Jinzhong 030801, China; 17835714706@163.com (Y.D.); ww1286687680@163.com (C.W.); lilitchi@outlook.com (L.L.); 15148745002@163.com (R.H.); 19722719701@163.com (X.S.); 15379367992@163.com (G.H.); wj13546605075@163.com (J.W.); m150356860511@163.com (M.N.); dong-s-q@163.com (S.D.); 2 Department of Basic Sciences, Shanxi Agricultural University, Jinzhong 030801, China; z1714770355@163.com; 3 Shanxi Institute of Functional Agriculture, Shanxi Agricultural University, Jinzhong 030801, China

**Keywords:** foxtail millet, balanced fertilization, soil nutrients, soil enzyme activity, soil microorganisms

## Abstract

The effects of balanced fertilization with nitrogen, phosphorus, and potassium (NPK) on foxtail millet productivity and the soil environment under the same conditions of total nutrients have received limited research attention. Therefore, in this study, three balanced fertilization patterns of 27-14-10 (T1), 27-17-7 (T2), and 30-10-11 (T3), and one no fertilization treatment (CK), a total of four treatments, were set up through a two-year field experiment to study the effects of balanced fertilization patterns on foxtail millet yield and soil environment. Mantel analysis was conducted to reveal the correlation between soil environmental factors and the community and their contribution to productivity. The results showed that: (1) all balanced fertilization treatments significantly increased foxtail millet yield, with the highest yield in the T1 treatment. (2) The contents of EC, available K, available P, and alkaline-hydrolyzable nitrogen in the soil of the two-year TI treatments were higher than those of the other treatments and increased by 7.20–9.36%, 24.87–52.35%, 55.83–56.38%, and 21.05–43.95%, respectively, compared with CK. (3) Soil urease activity in the T1 treatment increased significantly by 26.67% and 9.00% compared with the control over the two years. Sucrase activity increased by 36.27% and 23.88% in the T1 treatment compared to CK, and glutaminase activity increased by 33.33% and 19.23% in the T1 treatment compared to CK. (4) T1 treatment significantly increased the OUT number and diversity index of the soil bacterial community. (5) Mantel analysis and principal component analysis showed that available soil nutrients and soil enzymes were positively correlated, and soil enzymes and soil nutrients contributed more to foxtail millet productivity. In this study, the 27-14-10 balanced fertilization pattern was more effective, providing a theoretical basis for the research and development of special fertilizers for foxtail millet and offering technical guidance for realizing the light simplified cultivation of foxtail millet and sustainable development of cost–saving and increased efficiency.

## 1. Introduction

Foxtail millet (*Setaria italica*) is an ancient cereal crop with a cultivation history of 11,000 years in northern China [1]. China is the global leader in foxtail millet production, with its planting area accounting for more than 90% of the world’s total area [2]. As the leading crop of mixed grains in Shanxi, foxtail millet plays a key role in the current agricultural planting structure adjustment because of its characteristics such as low water consumption, drought and barren resistance, dual use of grain and feed, and deep-rooted agricultural civilization heritage [3]. Fertilization is an effective way to promote crop growth and development and improve crop nutrition levels, and a reasonable fertilizer ratio can not only promote the nutritional and reproductive growth of foxtail millet, but also save costs and protect the soil environment. Compound fertilizers, as integrated nutrient sources, play significant and multifaceted roles in agriculture and soil protection. Firstly, it provides a wide range of key nutrients required by crops, such as nitrogen, phosphorus, and potassium, which helps to maintain a balanced state of crop growth and enhance crop yields. Secondly, compound fertilizers can improve soil structure and promote the activities of beneficial microorganisms, thus enhancing soil fertility and biodiversity. Therefore, an in-depth study on the effects of compound fertilizers with different ratios of N, P, and K on foxtail millet yield and soil environment is of great significance to research on special foxtail millet fertilizers and realizing the light simplified cultivation of foxtail millet and sustainable development of cost-saving and increase efficiency.

Fertilizers play an important role in increasing crop yields in intensive agriculture in our country [4]. By 2050, the demand for food may reach twice the current demand, which indicates that fertilizer application remains an important agricultural activity for increasing crop yields [5]. A study has shown that there is a strong relationship between ten consecutive years of crop yield increase and fertilizer application [6]. An appropriate ratio of NPK can significantly improve crop growth and development, further promote nutrient uptake, and increase yield and economic benefits [7].

The effects of different fertilization regimes on soil microbial population numbers and community structure vary significantly [8]. Studies have shown that the application of compound fertilizers can effectively reduce the risk of nitrogen and phosphorus loss in the soil, and long-term fertilization can also improve nutrient utilization and enhance soil fertility [9]. Reasonable fertilization can also effectively improve soil physicochemical properties, and changes in soil fertility are determinants of changes in the structure of soil microbial communities, while indirectly affecting soil enzyme activities [10]. Ma et al. [11] showed through 50 years of nitrogen and phosphorus fertilization on the structure and function of soil microbial communities that nitrogen increased the α-diversity of archaea and that the β-diversity of archaea and bacteria, as well as soil function, were also mainly driven by nitrogen fertilization. Yang et al. [12] research showed that good fertilization management can improve soil nutrients and bacterial communities. Wu et al. [13] research showed that long-term application of nitrogen and phosphorus altered the key species composition of bacterial communities by affecting the nutrient content of rhizosphere soil, which shifted the pattern of community building from a stochastic to a deterministic process. Soil enzymes play a key role in soil nutrient cycling [14], and Wu et al. [15] research showed that nitrogen and phosphorus fertilizers can increase soil enzyme activity, thereby altering soil nutrient cycling.

Although previous studies have clarified the effects of compound fertilizer application on crop yield and soil environment, the content of nitrogen, phosphorus, and potassium in compound fertilizer is not the higher, the better, and the fertilizer demand characteristics of different crops are different, and should be combined with the fertilizer demand law of the crop, the reasonable allocation of nitrogen, phosphorus and potassium fertilizer to achieve the application fertilizers goal of “less fertilizer and high efficiency”. Under the condition of the same total nutrient content, there is a relative lack of reports on the effects of compound fertilizers with different ratios of N, P, and K on foxtail millet yield and the soil environment. Therefore, this experiment uses this as an entry point, with Zhangzagu 16 as the material, to investigate the impact of suitable nitrogen-phosphorus-potassium ratios in compound fertilizers on foxtail millet yield, soil nutrients, soil enzyme activity, soil bacteria, soil environmental factors, and the correlations between yield and bacteria, to establish high-yield and high-quality foxtail millet fertilizer formulations, and to screen the best combinations of NPK, in order to provide a theoretical base for the research and development of special fertilizers for foxtail millet, and offering technical guidance for realizing the light simplified cultivation of foxtail millet and sustainable development of cost-saving and increase efficiency.

## 2. Results

### 2.1. Effect of Different Compound Fertilizers on Foxtail Millet Yield

**Figure 1:**
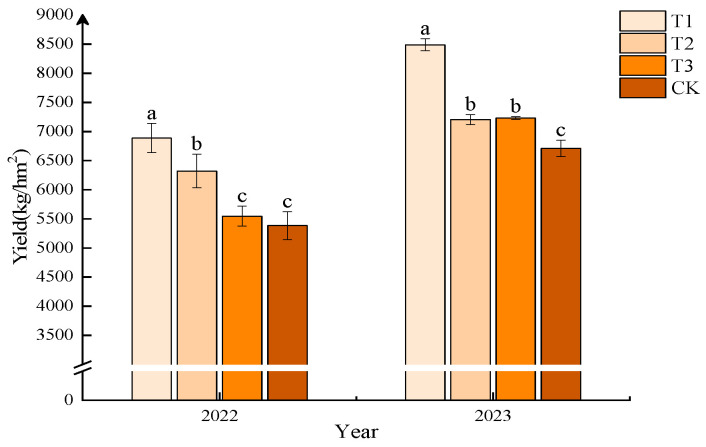
Effect of different compound fertilizers on foxtail millet yield. Note: Different letters in the figure indicate that the mean values of different treatments in the same year are significantly different (Tukey’s HSD test, *p* ≤ 0.05).

As shown in Figure 1, after two years of field trials, fertilization can increase the yield of foxtail millet. In both 2022 and 2023, the T1 treatment yielded the highest foxtail millet production, significantly higher than other treatments, with yields of 6886.63 kg/hm^2^ and 8487.58 kg/hm^2^, representing increases of 27.97% and 26.53% compared to the control group (CK), respectively. In 2022, T2 increased the yield by 13.99% compared to T3, but there was no significant difference in yield between T2 and T3 in 2023.

### 2.2. Effect of Different Compound Fertilizers on Soil Nutrients in Foxtail Millet

From Table 1, it is known that in 2022 and 2023, the soil EC content, available P, available K, and alkaline-hydrolyzable nitrogen contents were all the highest for the T1 treatment and significantly higher than those of the control treatment. Over the two years, the soil pH values in the fertilized treatments were lower than those in the non-fertilized treatment, indicating that a reasonable NPK fertilizer ratio can effectively alleviate soil alkalization. Compared to the control (CK), the total nitrogen content in the soil from fertilized treatments in 2022 significantly increased by 43.75%, 50.00%, and 51.56%, respectively, and in 2023, the soil total nitrogen content under the T1 treatment was the highest, increasing by 6.06% compared with CK. In 2022, the soil organic matter content under each fertilization treatment was significantly higher than that of CK, but there was no significant difference among the treatments in 2023.

### 2.3. Effect of Different Compound Fertilizers on Soil Enzyme Activities

**Figure 2:**
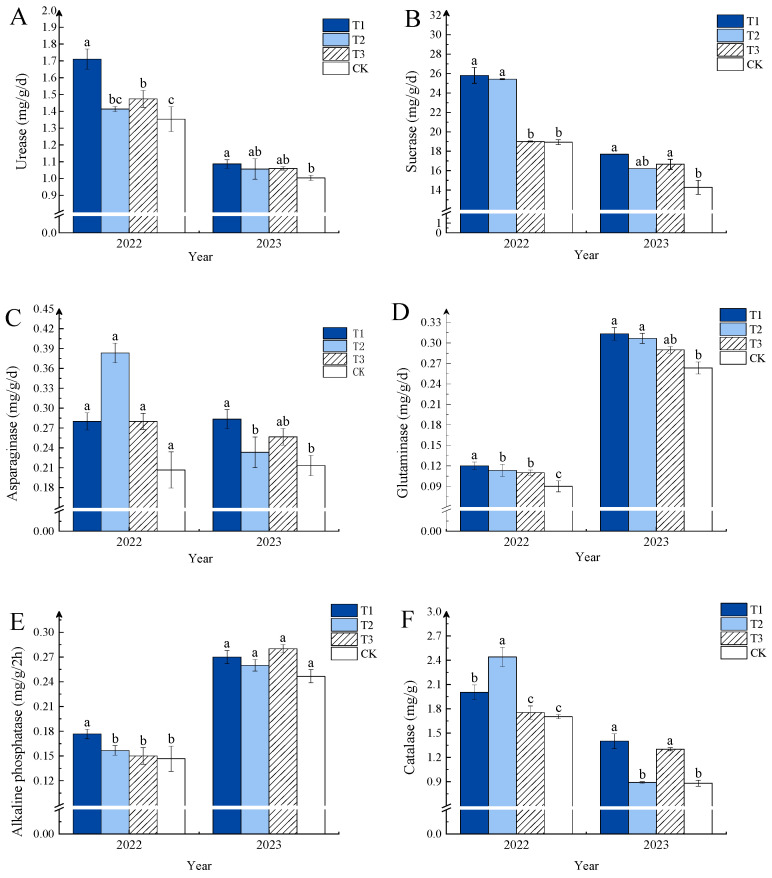
Effects of different compound fertilizers on soil enzyme activities. Note: Different letters in the figure indicate that the mean values of different treatments in the same year are significantly different (Tukey’s HSD test, *p* ≤ 0.05). (**A**) urease, (**B**) sucrase, (**C**) asparaginase, (**D**) glutaminase, (**E**) alkaline phosphatase, and (**F**) catalase.

From Figure 2, it can be seen that the effects of different NPK ratios of compound fertilizers on soil enzyme activity varied. In 2022 and 2023, the activities of soil urease (Figure 2A), sucrose enzyme (Figure 2B), and glutaminase (Figure 2D) were highest in the T1 treatment, significantly exceeding those of the control treatment. In 2022, the activities of asparaginase (Figure 2C) and catalase (Figure 2F) were highest in the T2 treatment, increasing by 80.95% and 43.53%, respectively, compared to CK, in 2023; however, the two enzyme activities of T1 treatment were highest, showing significant increases of 33.33% and 59.09% compared to the control. In 2022, alkaline phosphatase (Figure 2E) activity was the highest in the T1 treatment, showing a 20.00% increase over the non-fertilized treatment, while in 2023, there were no significant differences among the various treatments.

### 2.4. Effect of Different Compound Fertilizers on OUT Population of Soil Bacterial Communities

**Figure 3:**
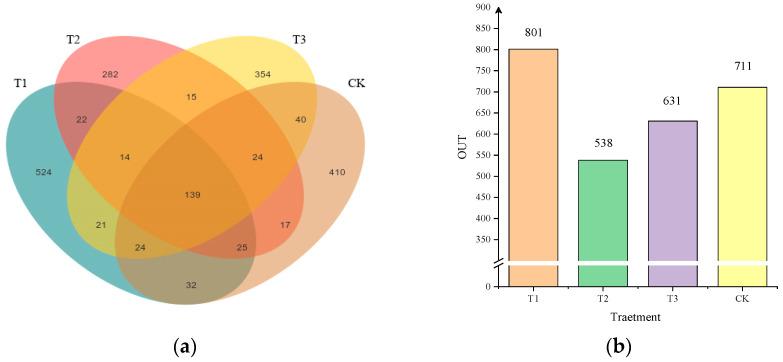
Effect of different compound fertilizers on OUT population of soil bacterial community.

After the application of different compound fertilizers, the soil bacterial community OUT (Operational Taxonomic Units) number changes, and the distribution Venn diagrams are shown in Figure 3. The total number of rhizosphere soil bacterial OUTs of different fertilizer treatments was 1943, and the total number of OUTs shared by each treatment was 139, which accounted for 7.15% of the total number of bacterial community OUTs, 19.55% of the unfertilized treatment, 17.35% of the T1 treatment, 25.84% of the T2 treatment, and 22.03% of the T3 treatment, respectively (Figure 3a). The highest number of OUT was found in the T1 fertilization treatment, which was 12.66% higher than the control treatment. The T1 fertilization treatment increased the number of soil bacterial exclusive species compared to the no fertilization treatment, while the T2 and T3 fertilization treatments decreased their numbers (Figure 3b). This indicates that the 27-14-10 ratio of compound fertilizer can increase the number of rhizosphere soil bacterial communities OUT in foxtail millet.

### 2.5. Effect of Different Compound Fertilizers on Soil Bacterial Diversity Indices

**Figure 4:**
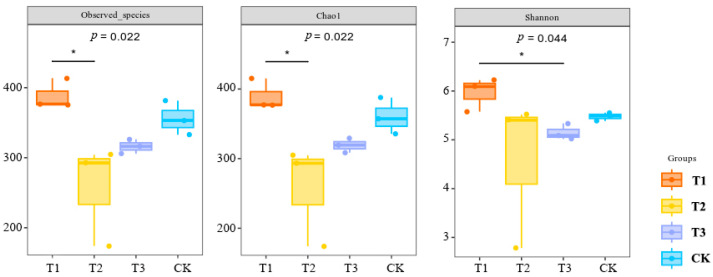
Effect of different compound fertilizers on the diversity index of soil bacterial communities (* indicates a significant correlation at the 0.05 level, *p* < 0.05).

The effects of different compound fertilizers on the observed species index, Chao1 index, and Shannon index of soil bacteria are shown in Figure 4. The observed species index is used to measure the number of different species observed in an area and the relative proportion of these species; the higher the index, the higher the diversity of species in the area and the healthier the ecosystem. Chao 1 index reflects the richness of microbial community; the higher the index, the higher the richness. The number of bacterial observed species and species richness of bacterial communities were lower in the T2 and T3 fertilization treatments compared with no fertilization treatment, and the observed species index and Chao1 index were the highest in the T1 treatment, which was significantly higher compared with those in the T2 treatment (*p* < 0.05). The Shannon index reflects the diversity of species; the higher the value, the higher is the diversity of species in the community. T2 and T3 treatments reduced the species diversity of the bacterial community compared to the control; the T1 treatment had the highest Shannon index and was significantly higher (*p* < 0.05) than the T3 fertilization treatment. This indicated that the 27-14-10 ratio of compound fertilizer could increase the species richness and diversity as well as the number of observed species in the rhizosphere soil bacterial community of foxtail millet.

### 2.6. Effect of Different Compound Fertilizers on the Composition of Soil Bacterial Communities

**Figure 5:**
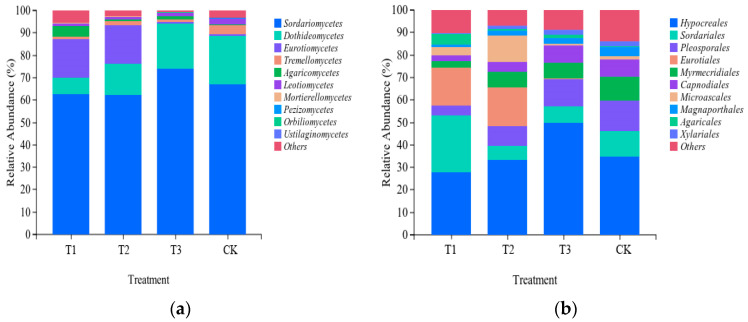
Effect of different compound fertilizers on soil bacterial community composition.

As can be seen from Figure 5, different fertilization treatments significantly affected the soil bacterial composition; at the level of Class (Figure 5a), T3 treatment increased the number of Sordariomycetes in the bacterial community by 10.45% compared to the control. The T3 and CK treatments had a higher number of Dothideomycetes than the T1 and T2 treatments, with an increase of 185.71% and 214.29%, respectively, compared to the T1 treatment. The Eurotiomycetes of the T1 and T2 treatments were significantly higher than those of the T3 and CK treatments. At the level of Order (Figure 5b), the Hypocreales were higher in T3 treatment than in other treatments, with a significant increase of 78.57% and 51.52% compared to T1 and T2 treatments, respectively. Sordariales were higher in the T1 treatment than in the other treatments, with an increase of 316.67% compared to T2. Pleosporales were significantly more abundant in the T3 and CK treatments than in the other treatments. Eurotiales were significantly higher in the T1 and T2 treatments than in the T3 and CK treatments.

### 2.7. Correlation Analysis of Soil Environmental Factors, Bacterial Community Composition and Yield

**Figure 6:**
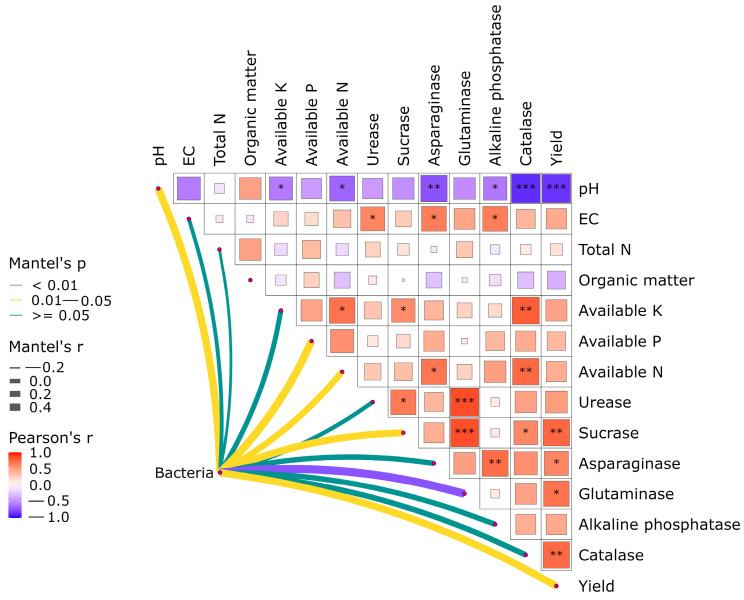
Correlation analysis of soil environmental factors, bacterial community composition, and yield. Note: EC is electrical conductivity, Total N is total nitrogen, Available K is available potassium, Available P is available phosphorus, and available N is alkaline-hydrolyzable nitrogen (*, ** and *** showed significant correlation at levels 0.05, 0.01 and 0.001, respectively).

The Mantel analysis of the soil bacterial community composition, soil chemical properties, soil enzymes, and yield is shown in Figure 6. Soil pH and soil enzymes exhibit a negative correlation, with a highly significant negative correlation between soil pH and catalase. Soil electrical conductivity is positively correlated with soil enzymes. Quick-acting nutrients show a positive correlation with soil enzymes, and available K and alkaline-hydrolyzable nitrogen were significantly positively correlated with catalase. Glutaminase shows a highly significant positive correlation with urease and sucrose. Yield is significantly positively correlated with sucrose and catalase, while it is highly significantly negatively correlated with pH. The bacterial community is highly significantly correlated with glutaminase.

### 2.8. Principal Component Analysis of Soil Environmental Factors, Bacterial Community Composition and Yield

**Figure 7:**
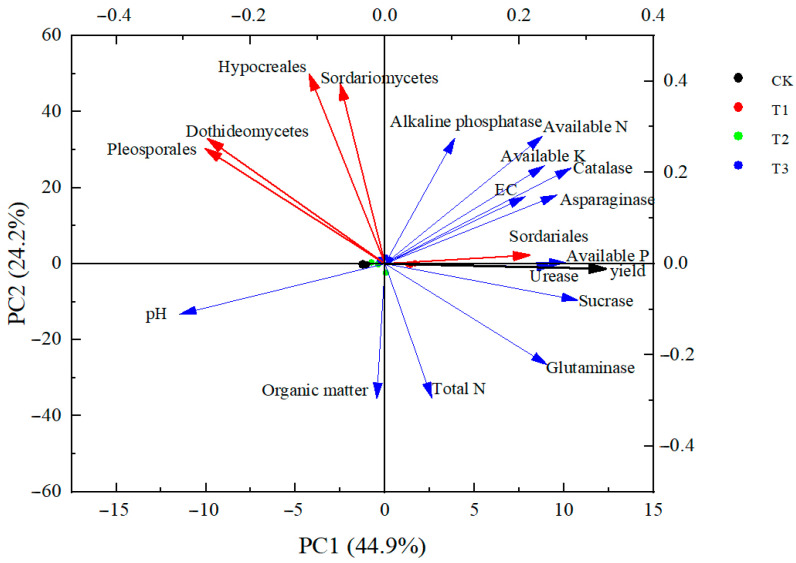
Principal component analysis of soil environmental factors, bacterial community composition, and yield. Note: EC is electrical conductivity, Total N is total nitrogen, Available K is fast-acting potassium, Available P is fast-acting phosphorus, and available N is alkali-dissolved nitrogen.

After correlation analysis, we found that there was a significant correlation between soil environmental factors, bacterial community composition, and yield, and PCA analysis could be performed. As shown in Figure 7, the first and second principal components explain 44.9% and 24.2% of the variation in the relationship between rhizosphere soil bacterial community composition, environmental factors, and yield, respectively. When the angle between soil environmental factors and bacterial community composition and yield was an acute angle, it indicated that they were positively correlated, and the smaller the angle of the acute angle, the greater the correlation, and vice versa for the obtuse angles. Soil environmental factors and soil enzymes contributed more to yield, and soil pH contributed the least, with available P, urease, sucrase, and Sordariales contributing the most to yield. A total of 12 principal components were extracted from the soil environmental factors, bacterial community composition, and yield indicators (as shown in Table 2), with the first principal component having an eigenvalue of 8.52, variance percentage of 44.85, and cumulative contribution rate of 44.9%. The second principal component has an eigenvalue of 4.6, variance percentage of 24.19, and cumulative contribution rate of 24.2%. The cumulative contribution rate of the first 11 principal components reaches 100%. Therefore, fertilizers suitable for foxtail millet growth should be selected in foxtail millet fertilization to provide an adequate nutrient supply environment for stable and increased foxtail millet production.

## 3. Materials and Methods

### 3.1. Overview of the Test Site

**Figure 8:**
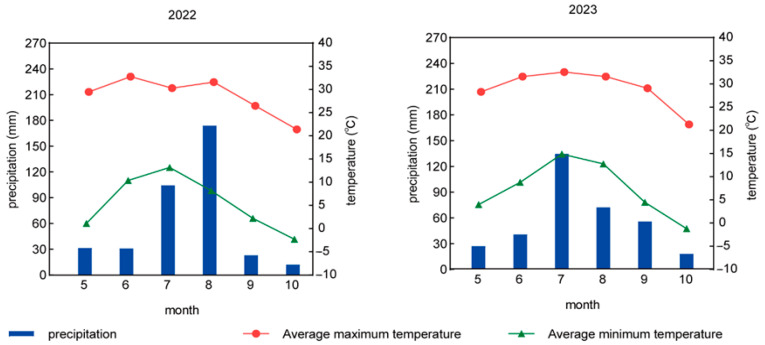
Meteorological data during the field experiment.

The experiment was conducted on 21 May 2022–1 October 2022 and 11 May 2023–3 October 2023 at the demonstration base of Hai Feng Farm and Ranch, Fanshi County, Xinzhou City, Shanxi Province (113°33′ E, 39°15′), with an elevation of 1105.87 m. The climate is a temperate continental monsoon climate, with an average annual temperature of about 6.3 °C, and average annual sunshine hours of 2906 h, annual precipitation of about 400 mm, frost-free period of 130 days, and effective cumulative temperature of 2–15 °C. The meteorological data obtained during the specific field experiment are shown in Figure 8.

The test site in 2022 was loamy soil, the test site in 2023 was sandy loam soil, and the previous crop in both years was corn. At the beginning of the experiment, initial soil samples were collected from the 0 to 20 cm surface soil profile, retrieved, air-dried naturally, and passed through a 1 mm sieve to remove impurities such as stones, gravel, leaves, and plant roots. The specific soil physicochemical properties are shown in Table 3.

### 3.2. Experimental Design

Before the experiment began, a survey was conducted on local foxtail millet varieties grown in Fanshi County, Xinzhou City, Shanxi Province. The results showed that Zhangzagu16 had the largest local promotion of the planting area. It is a new hybrid foxtail millet variety with strong cold and disease resistance, is capable of maintaining stable yields in relatively dry regions, and is best suited for local cultivation. Therefore, the foxtail millet variety used in this experiment was Zhangzagu16, selected and bred by the Zhangjiakou Academy of Agricultural Sciences and exclusively produced and operated by Hebei Xuntian Agricultural Science and Technology Co., Zhangjiakou, China.

Through this investigation, we determined the total nutrient content of the compound fertilizer widely used in the local area. Three different proportions of compound fertilizer (T1, T2, and T3) with the same local total nutrient content were set as the fertilization treatment, consisting of compound fertilizer N-P_2_O_5_-K_2_O (27-14-10) purchased from Chengdu Yuntu Agricultural Service Technology Co., Ltd., and compound fertilizer N-P_2_O_5_-K_2_O (27-17-7) from Anhui Liuguo Chemical Co., Ltd., and compound fertilizer N-P_2_O_5_-K_2_O (30-10-11) from Stanley Agricultural Group Co., Ltd., and no fertilizer was applied as a blank control (CK). A total of four treatments, 100 m^2^ per plot, were replicated three times. The application rate of each compound fertilizer was the same at 600 kg/hm^2^, surrounded by 1 m protection rows. Before sowing, all fertilizers were applied as basal fertilizers. The sowing was conducted in strip sowing with 1 film and 2 rows, 50 cm row spacing, and 6 cm plant spacing, and the other field management was the same as the local field management.

### 3.3. Measurement Methods

#### 3.3.1. Rhizosphere Soil Sampling Methods

The method of multi-point mixed samples was used to collect rhizosphere soil. At the maturity stage of the foxtail millet, 10 uniformly growing foxtail millet plants are randomly selected for each treatment. First, remove plant residues and other debris from the soil surface. Then, use a shovel to excavate the foxtail millet roots completely; the rhizosphere soil that was not tightly adhered to the roots was removed by the method of shaking the soil, and then the tightly adhered soil was collected with a sterile brush to serve as the rhizosphere soil for the test, that is, 10 sample points of rhizosphere soil. Next, place the 10 sample points of rhizosphere soil on a plastic sheet, break them up, and mix them thoroughly, spreading them into a square shape. Draw diagonal lines to divide the soil sample into four parts, then combine the two opposite angle parts into one portion, retaining one portion and discarding the other. If the sample obtained is still large, the quartering method can continue until the required number is reached. The retained rhizosphere soil sample is then placed in a self-sealing bag and labeled; that is, one rhizosphere soil sample is obtained. The above steps were repeated three times for each treatment, that is, three repetitions, and a total of 12 samples were collected for four treatments.

The rhizosphere soil of the plant was collected, and debris such as plastic, leaves, stones, and plant residues were removed, naturally air-dried indoors, ground, passed through a 1 mm sieve, and bagged for spare use. One part will be used for soil nutrient determination, while another part will be used for measuring soil enzyme activity.

#### 3.3.2. Soil Nutrient Determination Methods

The soil indexes were determined with reference to Soil Agrochemical Analysis [16], and the average value was taken after each treatment was repeated three times. According to the water-soil ratio of 5:1, after the determination of soil pH with a pH meter, the determination of electrical conductivity with a conductivity meter, the determination of soil total nitrogen content with a semi-micro Kjeldahl method, the determination of alkaline-hydrolyzable nitrogen with 1 mol/L NaOH alkaline hydrolysis and dispersion method, the determination of available P with 0.5 mol/L NaHCO_3_ method, the determination of soil organic matter with the potassium dichromate capacity method with external heating, the determination of available K with the leaching of NH_4_OAC, and the flame photometric method.

#### 3.3.3. Methods for Measuring Soil Enzyme Activities

##### Urease

Urease activity was analyzed using the sodium phenol-sodium hypochlorite colorimetric method [17]. Weigh out 5 g of air-dried soil and place it in a 50 mL Erlenmeyer flask, and add 1 mL of toluene. After 15 min, add 10 mL of 10% urea solution and 20 mL of citrate buffer (pH 6.7). Shake well and incubate at 37 °C for 24 h. Then, filter, transfer 3 mL of the filtrate into a 50 mL colorimetric tube, and add distilled water to a volume of 20 mL. Then, add 4 mL of sodium phenolate solution and 3 mL of sodium hypochlorite solution separately, adding them while shaking; after 20 min, allow the color to develop and add distilled water to a final volume of 50 mL. Within 1 h, perform the colorimetric measurement using a spectrophotometer at a wavelength of 578 nm. For each soil sample, substrate-free controls are set up, using 10 mL of citrate buffer (pH 6.7) instead of 10 mL of 10% urea solution. Other operations are the same as those for the sample experiments to verify the purity of the reagents and the decomposition of the substrate itself. Each batch of experiments sets up three soil-free blanks, meaning no soil samples are added, and other operations are the same as those for the sample experiments to eliminate the influence of the original NH_4_^+^-N in the soil samples on the experimental results. Prepare a standard series solution of NH_4_^+^-N: Accurately weigh 0.4717 g of ammonium sulfate (previously dried to a constant weight in an oven at 105 °C and cooled to room temperature in a desiccator) and dissolve it in deionized water to prepare a stock solution with a concentration of 1000 mg N L^−1^. Then, dilute this stock solution with deionized water to obtain a series of NH_4_^+^-N standard solutions with concentrations of 0, 0.2, 0.4, 0.8, 1.2, 1.6, 2.0, and 2.4 mg N L^−1^. The concentration of NH_4_^+^-N in the soil can be determined based on the NH_4_^+^-N standard curve, which allows for the calculation of urease activity in the soil. Soil urease activity is expressed as the amount of NH_4_^+^-N (in milligrams) produced per gram of dry soil within 24 h.

Urease activity = (C_1_ − C_2_ − C_3_) × V_1_ × 10^−3^ × (V_2_/V_3_)/(m × t) (1)

where C_1_, C_2_, and C_3_ represent the NH_4_^+^-N concentrations in the soil sample, substrate-free control, and soil-free blank, respectively; V_1_ is the color rendering volume; V_2_ is the culture solution volume; V_3_ is the volume of filtrate absorbed; m is the weight of dry soil; and t is the culture time.

##### Sucrase

Sucrase activity was determined by the 3,5-dinitrosalicylic acid colorimetric method [18]. Weigh 2 g of naturally air-dried soil was poured into a 50 mL conical flask, and 8% sucrose solution 15 mL, pH = 5.5 phosphate buffer 5 mL, and 5 drops of toluene were added sequentially. After shaking well, put into a constant temperature incubator and incubate at 37 °C for one day for 24 h. Remove at the end of incubation and filter quickly. Take 1 mL of the filtrate and transfer it into a 50 mL colorimetric tube. Add 3 mL of 3,5-dinitrosalicylic acid solution and then heat in a boiling water bath at 100 °C for 5 min. Afterward, cool the tube under running tap water for 3 min, and then dilute to a final volume of 50 mL with distilled water. Perform the colorimetric measurement using a spectrophotometer at a wavelength of 508 nm. To eliminate errors caused by the presence of sucrose and glucose in the soil, each soil sample needs to be subjected to a substrate-free control, and a soil-free control must be conducted for the entire experiment. Standard curve drawing: add 1 mg/mL of standard glucose solution 0, 0.1, 0.2, 0.3, 0.4, and 0.5 mL into the test tube. Distilled water is then added to reach a total volume of 1 mL. Next, 3 mL of 3,5-dinitrosalicylic acid solution is added and mixed thoroughly. The mixture is accurately reacted in a boiling water bath for 5 min. After that, the tubes are immediately cooled in cold water bath until they reach room temperature and then dilute to a final volume of 50 mL with distilled water. Perform the colorimetric measurement using a spectrophotometer at a wavelength of 508 nm. The standard curve is drawn with the OD value as the ordinate and glucose concentration as the abscissa. The activity of the sucrose enzyme is expressed as the amount of glucose (in milligrams) produced from 1 g of soil within 24 h.

Sucrase activity = (A_1_ − A_2_ − A_3_) × V_1_ × (V_2_/V_3_)/m (2)

A_1_, A_2_, and A_3_ were soil samples, no soil blank and no substrate control, and glucose mg were obtained by a standard curve; V_1_ is color rendering volume; V_2_ is culture solution volume; V_3_ is the volume of filtrate absorbed; m stands for the weight of dried soil.

##### Asparaginase

Determine asparaginase using the Kneyer’s reagent colorimetric method [19]. Weigh 5 g of naturally air-dried soil and place it into a 50 mL conical flask. Add 0.5 mL of toluene, and after 15 min, add 10 mL of 3% asparagine solution and pH = 6.7 phosphate buffer. After shaking thoroughly, place the conical flask in a constant temperature incubator and incubate at 37 °C for 1 day. After the incubation, add 30 mL of 1 N KCL solution to the mixture. Shake for 30 min and then filter. Take 5 mL of the filtrate and transfer it to a 50 mL colorimetric tube. Add 5 mL of 5% NaOH solution and 2 mL of 50% potassium sodium tartrate solution. Then, add 30 mL of distilled water, mix thoroughly, and finally add 2 mL of Kneyer’s reagent. Adjust the volume to 50 mL with distilled water. Let it stand for 10 min, and then measure the absorbance at 420 nm using a spectrophotometer. Each soil sample requires a substrate-free control, and the entire experiment must include a soil-free control. Standard curve preparation: Accurately weigh 0.3819 g of NH_4_CL and dilute it to a volume of 1 L. After diluting this solution tenfold, take 0.1, 0.3, 0.5, 0.7, 0.9, and 1.0 mL into a 50 mL volumetric flask and follow the steps mentioned earlier for colorimetric determination to create the standard curve. The activity of asparaginase is expressed as the amount of ammonia (in milligrams) released after 24 h from 1 g of soil.

Asparaginase activity = (A_1_ − A_2_ − A_3_) × V_1_ × (V_2_/V_3_)/m (3)

A_1_, A_2,_ and A_3_ were soil samples, no soil blank and no substrate control, and ammonia mg were obtained by a standard curve; V_1_ is color rendering volume; V_2_ is culture solution volume; V_3_ is the volume of filtrate absorbed; m stands for the weight of dried soil.

##### Glutaminase

Determine glutaminase using the Kneyer’s reagent colorimetric method [19]. Weigh 5 g of naturally air-dried soil and place it into a 50 mL conical flask. Add 0.5 mL of toluene, and after 15 min, add 10 mL of 3% glutamine solution. After shaking thoroughly, place the conical flask in a constant temperature incubator and incubate at 37 °C for 1 day. The post-cultivation steps are the same as those for determining asparaginase activity. Each soil sample requires a substrate-free control, and the entire experiment must include a soil-free control. The standard curve preparation is the same as that for asparaginase. The activity of glutaminase is expressed as the amount of ammonia (in milligrams) released after 24 h from 1 g of soil.

Glutaminase activity = (A_1_ − A_2_ − A_3_) × V_1_ × (V_2_/V_3_)/m (4)

A_1_, A_2,_ and A_3_ were soil samples, no soil blank and no substrate control, and ammonia mg were obtained by a standard curve; V_1_ is color rendering volume; V_2_ is culture solution volume; V_3_ is the volume of filtrate absorbed; m stands for the weight of dried soil.

##### Catalase

Catalase activity is determined using the potassium permanganate titration method [20]. Weigh 2 g of soil and place it in a conical flask. Add 40 mL of distilled water and 5 mL of 0.3% hydrogen peroxide solution. Shake at 120 rpm for 20 min. Add 5 mL of 3 mol/L sulfuric acid to stop the reaction filter. Then, take 25 mL of the filtrate and titrate it with 0.1 mol/L potassium permanganate solution to a light pink endpoint. The activity of catalase is expressed as the volume (in milliliters) of potassium permanganate consumed per 1 g of soil after 20 min.

Catalase activity = (A − B) × T (5)

A: the volume (in milliliters) of potassium permanganate consumed for titrating the soil filtrate; B: the volume (in milliliters) of potassium permanganate consumed for titrating 25 mL of the original hydrogen peroxide mixture; T: the correction value for the potassium permanganate titration.

##### Alkaline Phosphatase

Alkaline phosphatase activity is determined using the colorimetric method with disodium phenyl phosphate [20]. Weigh 5 g of soil into a conical flask, add 5 drops of toluene, followed by 20 mL of 0.5% disodium phenyl phosphate, and shake at low speed for 15 min. Then, incubate in a constant temperature incubator at 37 °C for 2 h. Filter, transfer 5 mL of the filtrate to a 50 mL volumetric flask, dilute with water to 20 mL, then add 0.25 mL of ammonium chloride-ammonium hydroxide buffer(pH = 9.8), 0.5 mL of 2% 4-aminoantipyrine solution, and 0.5 mL of 8% potassium ferricyanide solution. Color development and dilute with distilled water to 50 mL. Perform colorimetric measurement at 510 nm. Each soil sample requires a matrix-free control, and the entire experiment needs a soil-free control. Preparation of the standard curve: Take 0, 1, 3, 5, 7, 9, 11, and 13 mL of the phenol working solution, place them in 50 mL volumetric flasks, and add distilled water to a total volume of 20 mL. Then, add 0.25 mL of ammonium chloride-ammonium hydroxide buffer, 0.5 mL of 2% 4-aminoantipyrine solution, and 0.5 mL of 8% potassium ferricyanide solution. Finally, make up the volume to 50 mL and perform colorimetry at a wavelength of 510 nm within 15 min using a spectrophotometer. The activity of alkaline phosphatase is expressed as the milligrams of P_2_O_5_ per gram of soil after 2 h.

Alkaline phosphatase activity = (A_1_ − A_2_ − A_3_) × V_1_ × (V_2_/V_3_) × 0.32 × 2.29/m (6)

A_1_, A_2,_ and A_3_ were soil samples, with no soil blank and no substrate control, and P_2_O_5_ mg were obtained by a standard curve; V_1_ is color rendering volume; V_2_ is culture solution volume; V_3_ is the volume of filtrate absorbed; 0.32: the coefficient for expressing results in phosphorus units; 2.29: the coefficient for converting P to P_2_O_5_; m stands for the weight of dried soil.

#### 3.3.4. Soil Microorganisms

At the ripening stage of foxtail millet, the rhizosphere soil was collected and sieved through 80 mesh sieve; stones, plastics, leaves, plant stubs, and other debris were removed, mixed well, and then transferred into cryotubes and stored in liquid nitrogen for microbiological determination. The steps to be carried out in the experiment were executed according to the standard protocol provided by the Illumina platform through sample library construction, library quality inspection, library sequencing, and other steps. Library Construction: The process uses Illumina’s TruSeq Nano DNA LT Library Prep Kit to build the library. Firstly, the terminal repair process involves the excision of protruding bases at the 5′ end and the supplementation of missing bases at the 3′ end of the DNA using the End Repair Mix2 within the kit. Simultaneously, a phosphate group is attached to the 5′ end. The second step is the addition of an A at the 3′ end. In this process, the 3′ end of DNA is individually added to an A base to prevent the DNA fragments from self-connecting and to guarantee the connection of the DNA to a sequencing adapter with a protruding T base at the 3′ end. The third step is the addition of an adapter with a specific tag. This process is carried out to enable the DNA to ultimately hybridize to the Flow Cell. The fourth step entails the amplification of the DNA fragments that have been ligated with the adapter through PCR, followed by purification of the PCR system using BECKMAN AMPure XP beads. The fifth step involves the final fragment selection and purification of the library through 2% agarose gel electrophoresis. Library quality inspection: Take 1 μL of the library and conduct 2100 quality inspections of the library using the Agilent High Sensitivity DNA Kit on an Agilent Bioanalyzer machine. A qualified library should possess a single peak and no adapters. The Quant-iT PicoGreen dsDNA Assay Kit is employed on the Promega QuantiFluor for the quantification of the library. The calculated concentration of the qualified library should be greater than 2 nM. Library sequencing: For a qualified library, 2x250 bp paired-end sequencing is performed on the Illumina NovaSeq machine using the NovaSeq 6000 SP Reagent Kit (500 cycles). Firstly, the library intended for machine operation (with non-repeated index) is serially diluted to 2 nM and then mixed in accordance with the proportion of the desired data volume. The mixed library is denatured into single strands by 0.1 N NaOH for on-machine sequencing. The quantity of the library loaded onto the machine can be controlled within the range of 15–18 pM, depending on the actual circumstances. After sequencing, raw reads are obtained, and primer removal, quality filtering, denoising, splicing, and chimera removal are performed to obtain clean reads, which are spliced and assembled, coding genes are predicted, non-redundant gene sets are constructed, and non-redundant gene sets are subjected to functional annotation and taxonomic analyses in both general and special databases, and the composition and abundance information of the samples species are statistically determined. The data were analyzed on the genescloud platform (https://www.genescloud.cn/ accessed on 21 November 2023). Based on the results of species composition, abundance information, and functional annotation, species composition, alpha diversity, beta diversity, compositional differences, and correlations of soil environmental factors were analyzed among treatments. Three replicates were set up for each treatment, totaling four groups of 12 samples.

#### 3.3.5. Yield

At the foxtail millet maturity stage, uniformly growing plants were randomly selected from each plot with a sampling area of 4 m^2^. All selected foxtail millet ears were clipped and placed into mesh bags, naturally air-dried for threshing, weighed, and converted to hectare yield based on the area of each plot.

### 3.4. Data Processing

One-way analysis of variance (ANOVA) was performed using SPSS (20.0, IBM SPSS Inc., Chicago, IL, USA) software. Data and graphs were collated using Excel (2010, Microsoft, Redmond, WA, USA) software and plotted using Origin (2021, OriginLab, Hampden, MA, USA).

## 4. Discussion

### 4.1. Effect of Different Compound Fertilizers on Foxtail Millet Yield

The issue of food production security in China has always been a focus of people’s attention [21]. Producing more grain inside limited arable land is the first problem that needs to be solved, and fertilizer application is one of the most effective ways to increase crop yield. Foxtail millet absorbs large amounts of nitrogen, phosphorus, and potassium from the soil during the growth process. Among them, nitrogen directly affects the nutritional quality of foxtail millet and positively influences growth traits and yield [22]. Phosphorus indirectly affects the growth, development, and yield of foxtail millet by promoting photosynthesis and respiration and copes with phosphorus deficiency by increasing the growth of the foxtail millet root system [23]. In this study, after two years of field trial, it was shown that fertilizer application increased foxtail millet yield. In both 2022 and 2023, T1 treatment had the highest foxtail millet yield and was significantly higher than other treatments up to 6886.63 kg/hm^2^ and 8487.58 kg/hm^2^, which increased the yield by 27.97% and 26.53%, respectively, compared with CK. Meanwhile, the results of this experiment also showed that under the condition of the same total nutrient content, it is not that the higher the content of nitrogen, phosphorus, and potassium elements, the higher the yield. Liu et al. [24] research showed that reasonable nitrogen-phosphorus-potassium fertilizer application helps to increase crop yield, which is consistent with the results of the present study; it illustrates that if we want to achieve the increase in crop yield, it is necessary to find a proportion of nitrogen-phosphorus-potassium that is suitable for the needs of the crop itself. At the same time, nitrogen, phosphorus, and potassium fertilizers applied in conjunction with each other are better than a single use of one of the fertilizers in terms of disease and pest prevention and economic benefits.

### 4.2. Effect of Different Compound Fertilizers on Soil Nutrients

Nitrogen, phosphorus, and potassium nutrient content of soil, organic matter, and the EC value of soil are important factors in measuring the chemical properties of soil, and soil fertility is affected by the content of each of these indicators [25]. The organic matter in the soil is an important source of various nutrient elements required for plant growth, especially nitrogen and phosphorus. Organic matter also contains substances that stimulate plant growth, such as humic acid substances. In addition, organic matter makes the soil looser and creates a certain structure. Organic matter also has colloidal properties, which means that it is able to adsorb many cations; this property gives the soil fertility retention capacity and buffering properties [26]. In soil, nitrogen in its organic form can be divided into three types: semi-decomposed organic matter, microbial carcasses, and humus, which is predominantly humus. Most of the nitrogen in the organic form needs to be converted into the inorganic form through the transformation function of soil microorganisms so that it can be absorbed and utilized by plants. The present study showed that different compound fertilizers can increase soil nutrient content and reduce soil pH. This indicates that the application of compound fertilizers can not only lead to soil acidification but also improve the soil’s ability to retain nutrients, enhance its buffering capacity, loosen its structure, and create a favorable microenvironment for crop growth, which may ultimately result in more benefits than drawbacks. The results of previous research are consistent with those of this experiment, likely due to the application of a balanced proportion of nitrogen, phosphorus, and potassium compound fertilizers. Each nutrient plays a unique role in crop absorption and cannot be replaced; however, they can complement each other. For example, nitrogen fertilizers promote phosphorus absorption, while potash fertilizers enhance the effectiveness of phosphorus and improve the crop’s absorption and utilization of nitrogen.

### 4.3. Effect of Different Compound Fertilizers on Soil Enzymes

Soil enzymes are biologically active proteins present in soil and they play an important role in the metabolic processes of the soil. These enzymes are not only important indicators for assessing soil productivity and biological activity but also have irreplaceable functions in ecosystems [27]. The role of soil enzymes in ecosystems is primarily characterized by their regulation of nutrient cycling. These enzymes catalyze various biochemical reactions, facilitating the decomposition of organic matter and the release of inorganic nutrients, thereby supporting plant growth and nutrient cycling [28]. For example, urease, a specific enzyme, is widely present in the soil and can catalyze the hydrolysis reaction of urea to produce ammonia, carbon dioxide, and water, a process that is directly involved in the conversion of organic nitrogen. Sucrase is closely related to the content of nitrogen, phosphorus, and organic carbon in the soil and can generate glucose through a series of enzymatic reactions, increase the content of soluble nutrients in the soil, improve the biological activity of the soil, reflect the degree of soil maturity, and serve as the primary source of nutrients for plants and microorganisms, which is directly related to the growth of crops. Asparaginase is a key intermediate metabolite in the process of nitrogen cycling in soil and plays a crucial role in nitrogen transformation. By measuring the activity of asparaginase, we can understand the conversion of nitrogenous organic compounds in soil. Glutamine is a compound present in amino acids and accounts for a significant proportion of soil amino acids. Glutaminase is an enzyme capable of hydrolyzing the amide bond in glutamine and converting glutamine to glutamic acid and ammonia. Catalase, also known as contact enzyme, has an important role in the metabolic and respiratory processes of living organisms and also plays an important role class in the breakdown of secretions and residues of soil animals and plant roots. Alkaline phosphatase in the soil is able to hydrolyze organophosphorus compounds, thus increasing phosphorus availability and can reflect the level of soil fertility. This may be due to differences in soil maturity, which in turn could be influenced by variations in the levels of soluble nutrients present in the soil. In the present study, the activities of all the above soil enzymes were higher in the two years of the T1 fertilization treatment. This may be due to the more coordinated nitrogen, phosphorus, and potassium content of the T1 treatment. It can be seen that a reasonable allocation of NPK can increase the activities of various enzymes in the soil and help to further activate the nutrients in the soil, thus promoting soil maturation and crop growth and development. Additionally, changes in soil enzyme activity can reflect alterations in the soil microbial community structure and their response to environmental changes [29,30].

### 4.4. Effect of Different Ratios of Compound Fertilizers on Bacterial Community

Soil microorganisms are sensitive indicators of changes in climate and environmental conditions, and the structure and diversity of soil microbial communities reflect the quality of the soil to a certain extent. Li et al. [31] research showed that the application of nitrogen fertilizer reduced the alpha diversity of bacteria and increased the beta diversity and abundance of bacteria and that among the bacteria, there are primarily Actinobacteria, Alphaproteobacteria, Betaproteobacteria, Gammaproteobacteria, and Deltaproteobacteria. These microorganisms play a dominant and extensive role in the nitrogen metabolism process in soil. Ellen et al. [32] research found that N and P fertilizers applied in combination increased aboveground biomass, microbial biomass, and N cycling, as well as N, P, and C uptake and burial. This indicates that the comprehensive application of nitrogen, phosphorus, and potassium nutrients can improve the physical and chemical properties of soil, enhance soil fertility, and increase soil enzyme activity. Ma et al. [11] long-term fertilization experiments indicate that soil microbes are able to adapt to environmental changes caused by variations in soil nutrient stoichiometry not only through changes in microbial shifts in community composition and functional gene abundance but also the ability to regulate enzyme activities. In this study, it was found that the T1 treatment increased the number of OUTs in the bacterial community and improved the species richness and species diversity of the bacterial community and the number of species observations compared with no fertilization. In summary, nitrogen, phosphorus, and potassium nutrients can improve soil physicochemical properties, which in turn increases soil fertility and soil enzyme activities and creates a favorable environment for the life of soil bacteria.

In this study, Mantel analysis showed that there was a positive correlation between quick-acting nutrients and soil enzymes, and there was also an interaction between enzyme activity and bacterial community structure, such that a dynamic cycle was formed among the three. On the one hand, this may be due to the rational application of nitrogen, phosphorus, and potassium, which increases soil nutrients and enhances enzyme activity, thereby promoting the reproduction and growth of bacterial communities, resulting in increased diversity and abundance of bacteria [33]. On the other hand, it may be that the structure and composition of the bacterial community also influence the performance of soil enzyme activity. Different bacterial communities have different metabolic pathways and enzyme systems, and they have different degradation capacities for different substrates, which leads to differences in enzyme activities in the soil [34]. Soil enzymes, as key components involved in nutrient cycling, also have an extremely close relationship with changes in their activities and nutrient cycling. In summary, the mutually promoting relationship among the three factors is conducive to improving the overall fertility of the soil. At the same time, a rich bacterial community and sufficient soil enzymes can improve the ecological function of the soil, making it more resistant to external disturbances, such as pests, diseases, and extreme weather conditions. The interaction among these three promotes soil health and fertility, providing better conditions and guarantees for crop growth and development, thus improving crop productivity and quality, and achieving sustainable agricultural development.

## 5. Conclusions

This experiment investigated the effects of balanced fertilization with NPK on soil nutrients, soil enzymes, soil bacterial community diversity, and community structure under the same conditions as total nutrients. A reasonable ratio of NPK can increase soil nutrient content, decrease soil pH, increase soil enzyme activity, increase species richness and species diversity of the soil bacterial community, and increase foxtail millet yield. This study finally concluded that the 27-14-10 NPK balanced fertilization pattern had the best effect, which provided a theoretical basis for the research and development of special fertilizers for foxtail millet and technical guidance for the realization of the light simplified cultivation of foxtail millet and cost-saving and efficiency-enhancing agricultural sustainability.

However, the research results of this experiment have certain limitations. Firstly, climatic conditions can affect the fertilization effect. This study was conducted under specific temperate continental climate conditions, and the results need to be validated in different climates. Secondly, differences in soil types (such as organic matter content, pH, etc.) can also influence fertilization effectiveness; future research should consider multiple soil types. Additionally, seasonal variations significantly affect plant nutrient demand and absorption capacity, and long-term experiments can provide a more comprehensive assessment of the effectiveness of fertilization patterns. Lastly, dynamic changes in microbial communities are also important, and long-term monitoring of microbes is necessary to understand the impact of fertilization on the soil ecosystem. In summary, while the optimal fertilization pattern has been identified, practical applications should consider climate, soil characteristics, seasonal variations, and microbial dynamics to ensure the effectiveness and sustainability of fertilization.

## Figures and Tables

**Table 1:** Effect of different compound fertilizers on soil nutrients.

Year	Treatment	Electrical Conductivity	pH	Total Nitrogen (g/kg)	Organic Matter (g/kg)	Available K (mg/kg)	Available P (mg/kg)	Alkaline-Hydrolyzable Nitrogen (mg/kg)
2022	T1	90.80 ± 4.65 a	8.51 ± 0.04 b	0.92 ± 0.04 a	18.03 ± 0.44 a	146.99 ± 2.32 a	19.90 ± 1.02 a	61.31 ± 4.36 a
	T2	84.07 ± 2.31 ab	8.47 ± 0.04 b	0.96 ± 0.01 a	19.21 ± 0.20 a	128.44 ± 2.25 b	16.22 ± 0.41 b	46.10 ± 0.69 b
	T3	72.13 ± 11.23 b	8.48 ± 0.03 b	0.97 ± 0.12 a	18.78 ± 1.50 a	109.75 ± 0.07 c	16.71 ± 1.06 b	40.63 ± 1.54 b
	CK	83.03 ± 0.60 ab	8.79 ± 0.01 a	0.64 ± 0.18 b	13.53 ± 2.53 b	96.48 ± 4.61 d	12.77 ± 1.94 c	42.59 ± 5.86 b
2023	T1	98.33 ± 1.53 a	7.91 ± 0.04 c	1.05 ± 0.02 a	21.01 ± 0.46 a	89.83 ± 0.07 a	14.59 ± 1.64 a	59.22 ± 2.05 a
	T2	97.27 ± 3.36 a	8.10 ± 0.06 ab	1.04 ± 0.02 a	21.13 ± 0.72 a	67.17 ± 2.27 b	7.46 ± 0.31 b	46.82 ± 2.06 b
	T3	97.03 ± 3.00 a	8.06 ± 0.02 b	0.85 ± 0.07 b	18.21 ± 3.43 a	89.82 ± 3.99 a	8.59 ± 1.11 b	58.46 ± 5.71 a
	CK	91.73 ± 2.33 b	8.16 ± 0.01 a	0.99 ± 0.13 ab	21.82 ± 1.00 a	71.94 ± 5.96 b	9.33 ± 0.21 b	48.92 ± 1.89 b

Note: Values are means (*n* = 3) ± standard deviation. Different letters in the same column indicate significant differences in the mean values (Tukey’s HSD test, *p* ≤ 0.05).

**Table 2:** Principal component analysis of soil environmental factors, bacterial community composition, and yield.

Principal Component Number	Eigenvalue	Percentage of Variance (%)	Cumulative (%)
1	8.52172	44.85116	44.85116
2	4.59543	24.18647	69.03764
3	1.97325	10.38554	79.42318
4	1.53329	8.06996	87.49314
5	0.92284	4.85707	92.35021
6	0.47073	2.47751	94.82773
7	0.38478	2.02515	96.85288
8	0.25342	1.33381	98.18669
9	0.21478	1.13044	99.31713
10	0.0702	0.36949	99.68662
11	0.05954	0.31338	100
12	7.46 × 10^−29^	3.93 × 10^−28^	100

**Table 3:** Physical-chemical properties of the test soils.

Year	Total Nitrogen (g/kg)	Organic Matter (g/kg)	Alkaline-Hydrolyzable Nitrogen (mg/kg)	Available P (mg/kg)	Available K (mg/kg)	pH	Electrical Conductivity
2022	0.83	11.68	54.36	42.87	67.17	8.83	91.37
2023	0.35	9.45	42.27	13.29	159.1	8.27	89.21

## Data Availability

All data generated or analyzed in this study are included in this published article. For further information, please contact the corresponding author.
